# Community-level epidemiology of soil-transmitted helminths in the context of school-based deworming: Baseline results of a cluster randomised trial on the coast of Kenya

**DOI:** 10.1371/journal.pntd.0007427

**Published:** 2019-08-09

**Authors:** Katherine E. Halliday, William E. Oswald, Carlos Mcharo, Emma Beaumont, Paul M. Gichuki, Stella Kepha, Stefan S. Witek-McManus, Sultani H. Matendechero, Hajara El-Busaidy, Redempta Muendo, Athuman N. Chiguzo, Jorge Cano, Mary W. Karanja, Leah W. Musyoka, Tuva K. Safari, Lennie N. Mutisya, Idris J. Muye, Maureen A. Sidigu, Roy M. Anderson, Elizabeth Allen, Simon J. Brooker, Charles S. Mwandawiro, Sammy M. Njenga, Rachel L. Pullan

**Affiliations:** 1 Faculty of Infectious and Tropical Diseases, London School of Hygiene & Tropical Medicine, London, United Kingdom; 2 Eastern and Southern Africa Centre of International Parasite Control, Kenya Medical Research Institute, Nairobi, Kenya; 3 Faculty of Epidemiology and Population Health, London School of Hygiene & Tropical Medicine, London, United Kingdom; 4 School of Public Health, Makerere University, Kampala, Uganda; 5 Neglected Tropical Diseases Unit, Division of Communicable Disease Prevention and Control, Ministry of Health, Nairobi, Kenya; 6 Department of Health, County Government of Kwale, Kwale, Kenya; 7 Faculty of Medicine, Department of Infectious Disease Epidemiology, London Centre for Neglected Tropical Disease Research, School of Public Health, St Mary’s Campus, Imperial College London, London, United Kingdom; Ministère de la Santé Publique et de la Lutte contre les Endémies, NIGER

## Abstract

Most epidemiological studies of soil-transmitted helminth (STH) infections focus on school-going children. The majority of large-scale cross-sectional and longitudinal community-based studies have been conducted prior to the implementation of wide-scale mass drug administration (MDA). This study investigates age-related patterns in prevalence and intensity of STH infection, and associated risk factors, in a region of south coastal Kenya that had previously received three consecutive years of school-based deworming (2012–14) and four rounds of community-based MDA for lymphatic filariasis between 2003 and 2014. Between March and May 2015, a cross-sectional survey was conducted in 120 community clusters as a baseline for a cluster randomised trial. Individuals aged two years and above were randomly selected during household surveys and requested to provide stool samples, which were assessed for presence and intensity of STH using the duplicate Kato-Katz thick smear method. Species-specific predictors of presence and intensity were investigated through multilevel logistic regression and zero-inflated negative binomial regression models. Of the 19,684 individuals who provided a stool sample, 21.5% were infected with at least one STH. Hookworm was the predominant species, with an overall prevalence of 19.1%; *Trichuris trichiura* prevalence was 3.6% and *Ascaris lumbricoides* was negligible (0.4% prevalence). The vast majority were light intensity infections. Prevalence and intensity of hookworm infection were significantly higher in adults and males, and were associated with environmental conditions, low socio-economic status, household flooring, individual and household water, sanitation and hygiene (WASH) characteristics and behaviours, previous treatment, lack of shoe-wearing and not attending school. In contrast, *T*. *trichiura* was more commonly found in school-aged boys and those living in communities close to the coast, with reduced infection in the least poor individuals with private latrine access. Overall, results show that despite several years of school-based deworming, hookworm infection remains common among untreated adults in this population, suggesting that this strategy alone is insufficient to reduce community-wide hookworm infection and in the longer term to eliminate transmission.

**Trial registration:**

ClinicalTrials.gov NCT02397772

## Author summary

Soil-transmitted helminth (STH) infections, including *Trichuris trichiura*, *Ascaris lumbricoides* and the hookworms *Ancylostoma duodenale* and *Necator americanus*, remain endemic in many regions of sub-Saharan Africa (SSA), including parts of Kenya. The current WHO-recommended treatment strategy focuses on morbidity control, and comprises periodic deworming of population groups at particular risk, including pre-school and school–age children. Consequently, the majority of epidemiological descriptions of STH have focused on infection in this age group, and are conducted using a school platform. There is therefore a notable lack of age-stratified data (that includes adults) from communities. We present data from a community-wide, cross-sectional survey of STH infection across 19,684 individuals and investigate associated risk factors at the household and individual level. We demonstrate highest prevalence and intensity of hookworm (the predominant species in this setting) in adults and males—a group not routinely included in deworming activities. There was marked geographic variation in infection risk across the study area, and an enduring relationship between infection risk and factors associated with poor access to sanitation and hygiene. Conducted after three years of ongoing, annual school-based deworming, the findings presented here are likely to be representative of many regions of SSA, with similar epidemiological and demographic profiles, implementing the current WHO-recommended STH control strategy.

## Introduction

Periodic large-scale administration of anthelmintic medication to populations at risk can effectively reduce the disease burden of soil-transmitted helminth (STH) infections [1], which together are responsible for an estimated 4.98 million years lived with disability each year [2]. For this reason, the World Health Organization (WHO) has set the goal for STH as—control of morbidity attributable to infection, with a treatment coverage target of 75% of pre-school and school-age children living in areas of risk by 2020 [3]. Countries are rapidly progressing towards this goal: by 2016, preventive chemotherapy programmes were delivering a reported 947.9 million benzamidazole treatments globally, either as part of LF elimination programmes (495.6 million treatments) or as school-based deworming (452.3 million treatments) [1]. The global community is increasingly looking beyond these targets to ask whether transmission interruption is possible [4–7], with several large-scale impact evaluations currently examining the impact of mass deworming targeting all ages [8–10]. However, there are few comprehensive contemporary evaluations of STH epidemiology in communities that have undergone repeat rounds of mass drug administration, with the majority of studies focusing on school children. In SSA, for example, only 10% of the STH surveys available through the Global Atlas of Helminth Infection included adults and the majority of these were conducted prior to large-scale intervention [11, 12]. A similar pattern is seen for Asia [13].

To understand how best to reduce STH transmission, it is essential to have age-stratified epidemiological data, as these can highlight age groups among whom infection remains common, most intense and therefore serve as a reservoir to re-infect the treated age groups. In this study, we present data from a large-scale cross-sectional survey of STH infection and intensity conducted in 2015 in Kwale county, Kenya, which has benefitted from consecutive, annual rounds of deworming implemented by the Ministry of Health and Ministry of Science Education and Technology through the National School Based Deworming Programme (NSBDP) since 2012 [14]. The dataset used represents the baseline parasitological survey for the TUMIKIA trial, an evaluation of school- versus community-wide annual and biannual deworming conducted between 2015 and 2017 [8, 15]. The aim of the present analysis is to describe the population distribution of STH species infection and investigate associated environmental, household and individual risk factors.

## Methods

### Study setting and population

The survey was conducted in Kwale County, Kenya. Kwale is an environmentally heterogeneous area, comprising four subcounties, with a total population of approximately 760,897 [16]. As a county, Kwale demonstrates high levels of income inequality and low levels of access to water and sanitation. The region is primarily rural, with many communities living on subsistence farming of maize and cassava.

The predominant STH species is hookworm, with *T*. *trichiura* and *A*. *lumbricoides* found to a lesser extent [17, 18]. The county has received a number of anthelmintic interventions. Annual school-based deworming with albendazole has been implemented successfully since 2012 (with a prior round in 2009) with programmatic coverage of 82% of pre-school and school-age children (2–14 years) in 2014 [19, 20]. Additionally, there have been four rounds of community-based mass drug administration (MDA) for lymphatic filariasis (LF), in which albendazole and diethylcarbamazine (DEC) were delivered to individuals aged 2 years and above. These LF MDA rounds were implemented in Kwale County in 2003, 2005, 2008 and 2011. For the two most recent MDA rounds prior to this survey, 2008 and 2011, programmatic coverage levels of 62.7% and 58.3% were reported [11, 20, 21]. For the ten years prior to this survey, Child Health Days (Malezi Bora) have been implemented on a biannual basis, targeting children under five years with benzimidazoles—either albendazole or mebendazole—and vitamin A [22]. However, coverage has been low, with surveys indicating as few as 19.6% of young children (aged 12–59 months) were dewormed through this programme in 2012 [22]. Finally, national guidelines state pregnant women are to receive benzimidazoles during their antenatal clinic visits from the second trimester.

### Survey design and recruitment

This survey constitutes the baseline survey for the TUMIKIA trial, a cluster randomised evaluation of the impact of alternative treatment strategies on the prevalence and intensity of STH infection. The sample size determination for the trial outcomes is detailed by Brooker *et al* (2015) and resulted in the selection of 225 individuals in each of 120 clusters [8].

**Fig 1:**
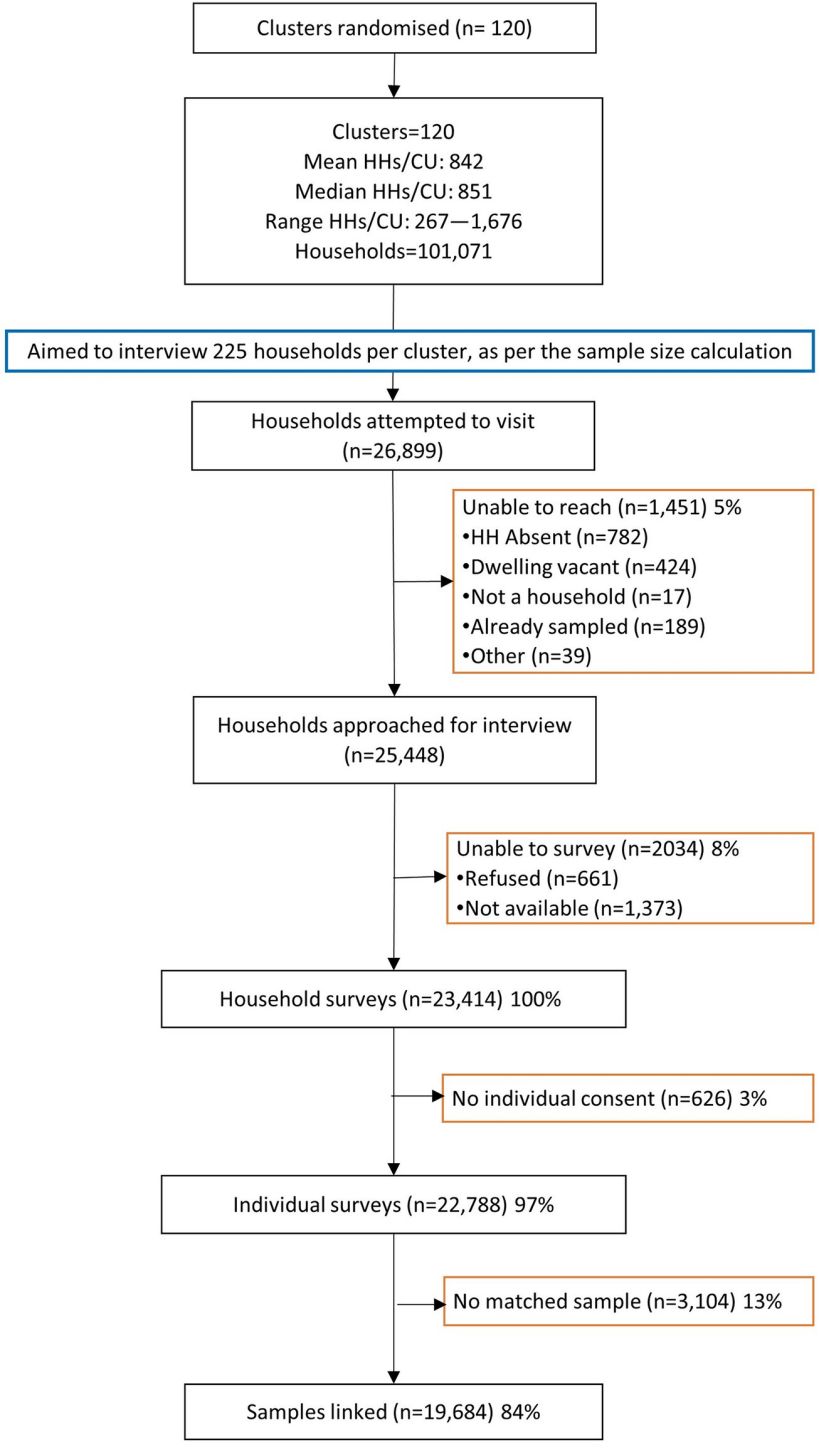
Data flow diagram. Data included for the household and individual-level surveys as well as the parasitological sample collection and analyses conducted for individuals across the 120 clusters on the south coast of Kenya, 2015. Households (HHs) Community Units (CUs).

**Fig 2:**
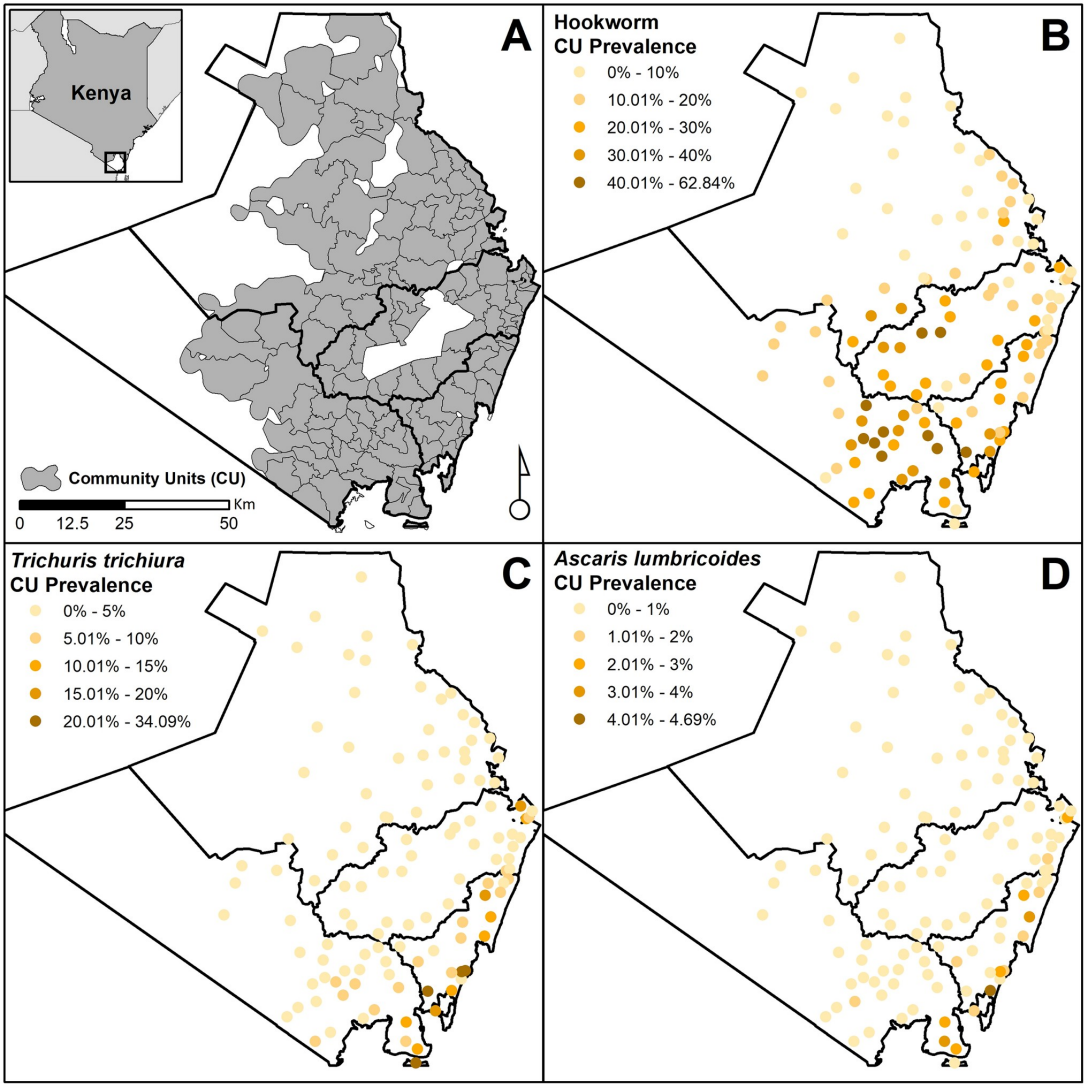
(A) The 120 study clusters (community units) in Kwale County, south Kenya, 2015 inset depicts the location of Kwale County within Kenya. Also shown are the cluster-level geographical distributions of (B) hookworm infection, (C) *T*. *trichiura* infection, (D) *A*. *lumbricoides* infection. Points depict the centroids of each cluster. Note that the map legend scale differs for each species.

An initial census of households in Kwale County was used to delineate community clusters, broadly synonymous with Ministry of Health (MoH) Community Units (CU), a health-service delivery structure covering a population of approximately 1,000 households. Of 130 clusters delineated across Kwale County, 120 were included in the survey (Figs 1 and 2a) comprising a population of approximately 540,000. Sample clusters comprised between 1 and 24 villages (median 7), and cluster size varied between 267 households (an island) and 1676 households (an urban setting) with a median of 851.

In each cluster, 225 households were selected using simple random sampling, regardless of cluster size, with an additional 75 households selected to replace refusals or migrated households. This sample size and approach was based on the trial design, powered to compare the primary outcome across arms. At each household, structured questionnaires were conducted with heads of household or primary caregiver on smartphones using SurveyCTO software (Dobility, Inc., dobility.com). The questionnaire manual, pdf and electronic form can be downloaded via this link: https://www.lshtm.ac.uk/research/centres-projects-groups/laser#tools--training. Household locations were collected in World Geodetic System 1984 datum using the smartphones’ Global Positioning System (GPS). In each sampled household, all household members were first enumerated along with their demographic information and education status, and a randomisation function then selected an individual to provide a stool sample. Eligibility criteria included: individuals 2 years old and above, who provided informed consent (and assent where appropriate). If the first sampled individual was not present and could not be located or was unwilling or unable to provide a stool sample, the form selected a replacement individual, up to a total of three per household. Observed household construction materials and reported household assets were collected to derive a measure of socioeconomic status (SES). Information on reported household access to water, sanitation, and hygiene (WASH) facilities was collected, and structured observations were made of sanitation and hygiene facilities. Individual-level information collected from the member providing the stool sample included WASH-related behaviour, observed shoe wearing and reported history of deworming. Questionnaire data were uploaded from the smartphones daily. Assembled questionnaire data were linked to parasitology results using a unique identifier. Discrepancies and queries during linkage were verified against the original laboratory books.

### Parasitological assessments

Stool samples were transported to a nearby health facility laboratory and examined in duplicate within one hour of processing, using the quantitative Kato-Katz (KK) thick smear method (41.7mg templates). Duplicate slides prepared from the single day stool samples were read by independent microscopists and a 10% quality control check was performed by a supervisor. Egg counts were enumerated for each species separately. The outcomes of interest were presence and intensity of infection with hookworm, *T*. *trichiura* and *A*. *lumbricoides*. Infection was defined based on presence of at least one egg across the duplicate slide readings, and intensity was expressed as the arithmetic mean of eggs per gram (epg) of faeces across the two slides. Infection intensities were categorised according to the WHO classification; for hookworm light (1–1,999 epg) moderate (2,000–3,999 epg) and heavy (>4,000 epg); for *T*. *trichiura* light (1–999 epg) moderate (1,000–9,999 epg) and heavy (>10,000 epg); for *A*. *lumbricoides* light (1–4,999 epg) moderate (5,000–49,999 epg) and heavy (>50,000 epg) [23].

### Environmental measures

A suite of environmental and topographic datasets were explored as potential environmental drivers of STH in the study area. Further details are provided in supplementary information (S1 Text, S1 and S2 Figs) but in brief, maps of Enhanced Vegetation Index (EVI) and Land Surface Temperature (LST) were produced by processing satellite images provided by the Moderate Resolution Imaging Spectroradiometer (MODIS) instrument operating in the Terra spacecraft (NASA) at a resolution of 250m [24, 25]. The fortnightly continuous gridded maps were aggregated by calculating the mean for the period of study. We obtained raster datasets of elevation and aridity at 1km^2^ from the Consortium for Spatial Information (CGIAR-CSI) [26]. Estimates of soil acidity (pH KCl) and sand content were extracted from soilgrids.org at a resolution of 250m [27]. Estimates of population density for 2015 were obtained from the WorldPop project, which was used to classify areas as urban, peri-urban or rural areas [28]. The range of environmental and topographic data were extracted using point-based extraction for each household using ArcGIS 10.3 (Environmental Systems Research Institute Inc. Redlands, CA, US). Households without GPS coordinates were given the village mean or mode value for continuous and categorical environmental measures, respectively. Continuous variables were categorised by tertiles for analysis.

### Analysis

Data management and analyses were performed using STATA version 14.0 (STATA Corporation, College Station, TX, USA). Prevalence and intensity descriptive analyses for the three STH species were calculated using robust standard errors to allow for clustering.

A factor analysis was used to determine the relative SES of households surveyed, using dichotomous indicators of ownership of a motorbike, bicycle, mobile phone, radio, television, electricity, sofa set and household wall and roof materials. Factor analyses were performed separately for rural and urban/peri-urban households, because of the difference in distribution of these variables in relation to SES between these distinct settings. The indices were divided into quintiles within each setting and subsequently the second, third and fourth quintiles were combined (middle) prior to analysis, with the first (poorest) and fifth (least poor) indicating the relative wealth extremes. During analysis the poorest and least poor groups were compared against the middle group, taken as the reference group.

Risk factor analyses were conducted for hookworm and *T*. *trichiura* infection. *A*. *lumbricoides* infection was not modelled due to very low infection levels found in the study population. Population-averaged models were used to estimate associations for both presence and intensity of infection. Univariable associations between presence of infection and individual-, household- and environmental-risk factors were assessed using generalised estimating equations (GEE), assuming a binomial probability distribution, and an exchangeable correlation structure, accounting for clustering at the community unit (cluster) level [29]. Associations between STH intensity (epg) and risk factors were modelled using a zero-inflated negative binomial regression [30] of egg counts, with quantity of stool assessed per sample included as an offset. Quantity of stool assessed was calculated according to the template size (41.7mg) and number of slides prepared and read per sample. The model accounted for clustering at the community unit (cluster) level using a clustered sandwich estimator. Both eggs per sample and egg output per person were well described by the negative binomial distribution with variance much greater in value than the mean across faecal samples and across people. Variables in the inflation model were decided *a priori* as age, sex and aridity, with aridity being used as an indicator of environmental suitability for STH transmission [31]. *A priori* interactions between age and sex were investigated in the prevalence models for both species. We used a backwards stepwise strategy to build separate multivariable models for prevalence and intensity of both species.

Separate subgroup analyses were conducted to estimate the relationship between both hookworm and *T*. *trichiura* infection and observed sanitation conditions in those households with facilities, using GEE, assuming a binomial probability distribution, and an exchangeable correlation structure, accounting for clustering at the community unit (cluster) level and adjusting for SES.

### Ethical considerations

The study was approved by the Kenya Medical Research Institute (KEMRI) Scientific and Ethics Review Unit (SERU No. 2826) and the London School of Hygiene & Tropical Medicine (LSHTM) Ethics Committee (#7177). Prior to the survey, stakeholders’ meetings were held at the national, county and sub-county levels, after which sensitization meetings were held with chiefs and assistant chiefs from all locations and sub-locations. The 880 villages included in the TUMIKIA Project were sensitized through a total of 583 village meetings. Written informed consent was sought from the household head or adult answering the household-level questionnaire, and consent was also sought from the individual selected to provide the stool sample and complete the individual-level questionnaire. Parental consent was sought for participants between 2 and 17 years and written assent was additionally obtained from children aged 13 to 17 years. All information and consent procedures were conducted in Kiswahili.

## Results

### Sample population

Of the 25,448 households initially approached for interview, 661 (2.6%) households refused and 1,373 (5.4%) were unavailable at the time of the household visit (Fig 1). Among households where a survey was conducted, in 626 (3.0%) households the individuals selected to provide samples did not provide consent. The individuals for whom a sample could not be matched (n = 3104) were very similar to the final individuals included in the analyses with respect to demographic and economic characteristics (S3 Table).

**Fig 3:**
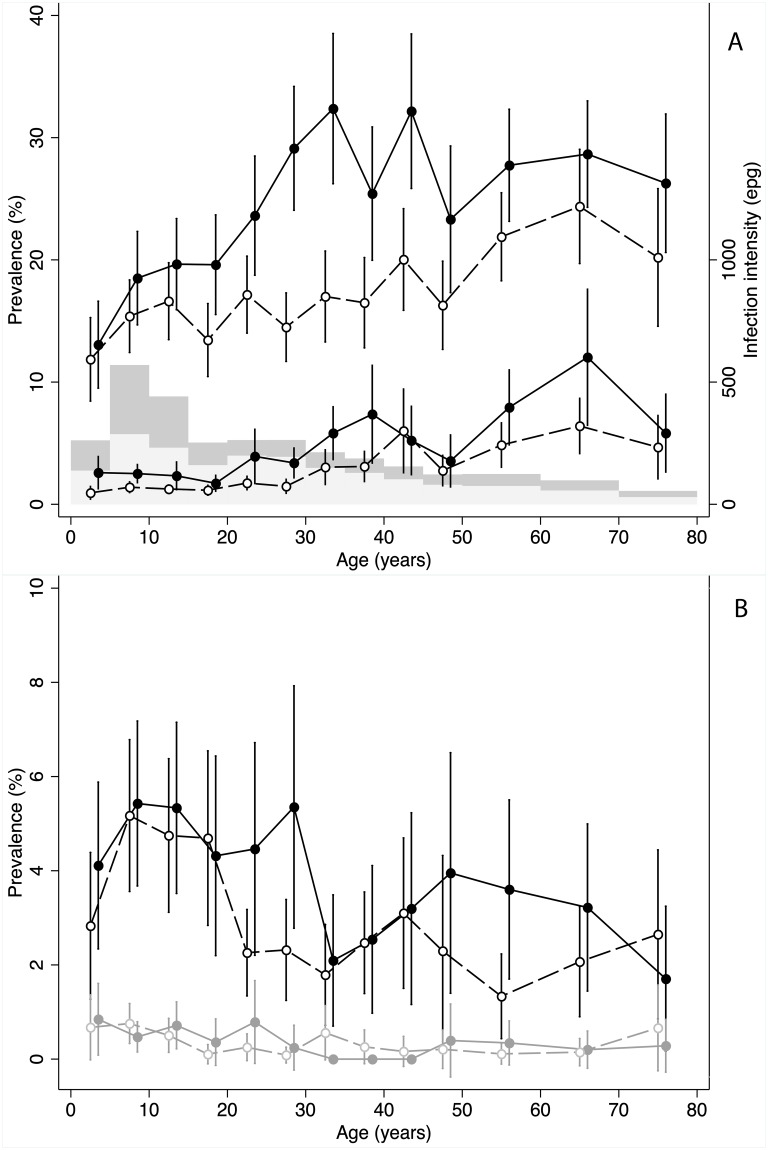
Age-infection profiles for STH species. (A) Prevalence (upper lines) and intensity (lower lines) of hookworm infection by age for males (solid line and circles) and females (dashed lines and empty circles). The histogram indicates the proportion sampled in each age-group by sex [males dark grey and females light grey]. (B) Prevalence of *T*. *trichiura* (black) and *A*. *lumbricoides* (grey) by age for males (solid line and circles) and females (dashed lines and empty circles). Intensity not depicted for *T*. *trichiura* and *A*. *lumbricoides* due to low infection prevalence.

A total of 19,684 individuals aged 2 years and above gave consent (and assent where required) and provided stool samples, which were linked with household- and individual-level information, and are included in the analyses. Median age was 22 years (inter-quartile range, IQR: 9–40) and the male:female ratio was 0.67, due to under-sampling of adult males, particularly in the 20–40 year age group (Fig 3a).

### STH infection by species

Overall, 21.5% of sampled individuals were infected with an STH infection (15.6% in under 5s, 20.9% in 5–14 years and 22.5% in ≥15 years) and 2.2% had moderate-high intensity (MHI) infections (1.8% in under 5s, 1.6% in 5–14 years and 2.5% in adults). Only 7.1% (n = 301) of infected individuals harboured multiple STH species. Initial observations of graphical outputs highlighted two outliers, one adult female with a hookworm intensity of 137,460 epg and one male <5 years with a *T*. *trichiura* intensity of 99,804 epg. These were subsequently excluded for the following descriptions and risk analyses.

Prevalence of hookworm infection was 19.1% (95% confidence interval [CI]: 16.4–21.7%); 12.5% in under 5s, 17.4% in 5–14 years and 20.7% in adults; with over 90% (3,394/3,750) of infected individuals exhibiting light infections (< = 1,999 epg). The arithmetic mean hookworm intensity was 161 epg (standard deviation [SD]: 1,083), and 843 epg (SD: 2,363) in those infected. Hookworm-infected individuals were found in 119 of the 120 clusters although considerable heterogeneity in prevalence by cluster was observed (Fig 2b); 16 clusters exhibited a hookworm prevalence below 5%, whereas hookworm prevalence exceeded 50% in three clusters.

A total of 710 (3.6%) individuals were infected with *T*. *trichiura*, with prevalence of infection varying markedly by cluster. In the 93 clusters in which infection was observed, prevalence ranged from 0.5% to 34.1%. *T*. *trichiura* infection was focal, localised to the coast, with the highest infection prevalence found on an island (Fig 2c). Again, infections were predominantly low intensity, with an arithmetic mean intensity of 12 epg (SD: 219) and a mean intensity of 329 epg (SD: 1,109) in those infected. *A*. *lumbricoides* infections were rare (0.4%) across the study area, with only 78 infections found across 33 clusters (Fig 2d), and of moderate intensity in those infected (on average 9,387 epg (SD: 18,542).

### Demographic infection profiles by species

Hookworm prevalence increased with age across both sexes, and was higher in males (Fig 3a). Infection intensity remained fairly constant through childhood, before increasing at age twenty and above in males, and thirty and above in females. *T*. *trichiura* infection prevalence and intensity were greatest in the younger age groups, and again in males. *A*. *lumbricoides* displayed no clear age or sex patterns in prevalence (Fig 3b) and given the very low levels was not examined further.

### Relationship between community-level prevalence and intensity

**Fig 4:**
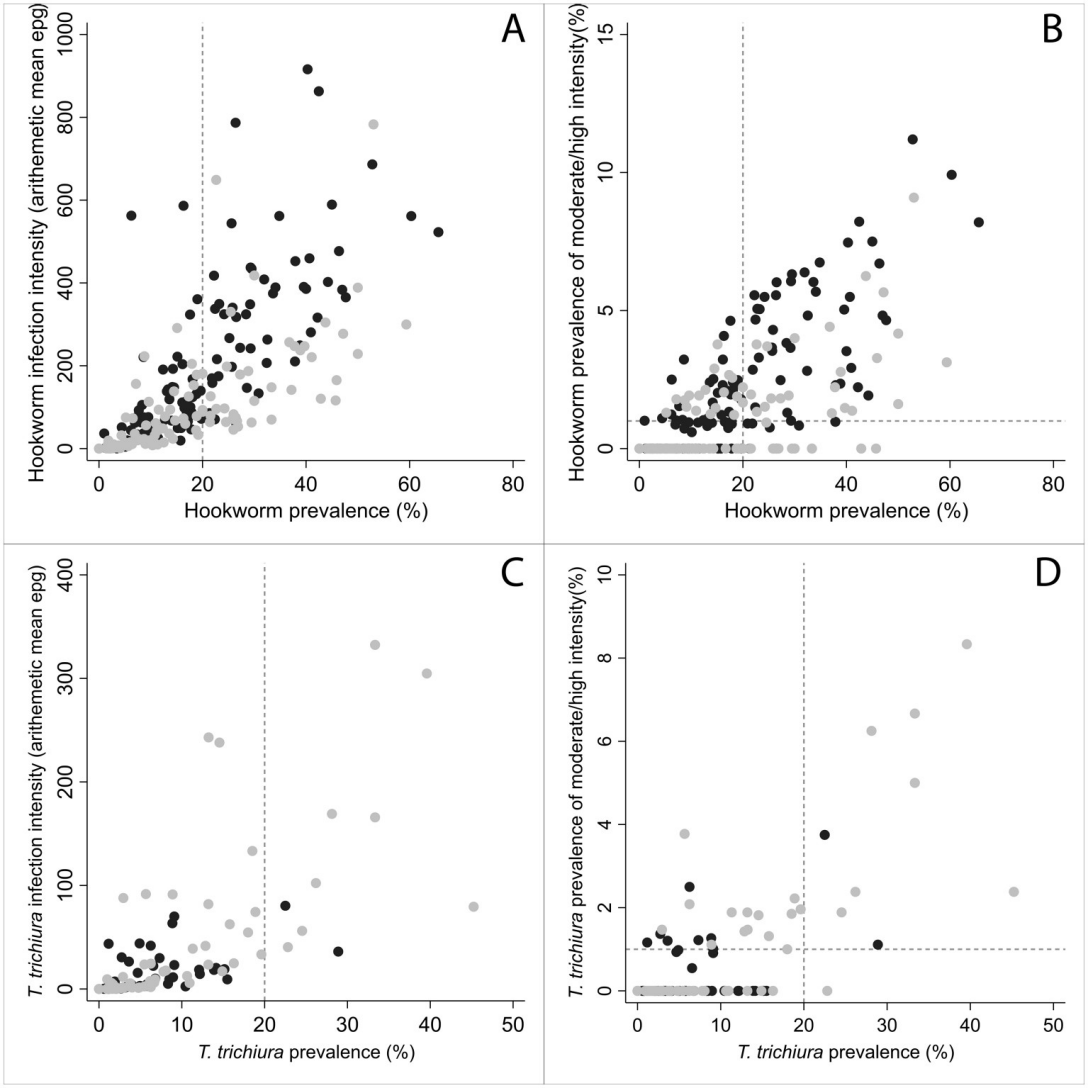
The relationship between cluster prevalence and intensity for hookworm and *T*. *trichiura* for both children (2–14 years) and adults (≥15 years) separately. (A) Relationship between prevalence of hookworm infection and arithmetic mean intensity for children (grey circles) and adults (black circles) (B) Relationship between prevalence of MHI infections and prevalence of hookworm infection in children (grey circles) and adults (black circles) [outlier of 137,460 epg excluded]. (C) Relationship between prevalence of *T*. *trichiura* infection and arithmetic mean intensity for children (grey circles) and adults (black circles) (D) Relationship between prevalence of moderate and high intensity (MHI) infections and prevalence of *T*. *trichiura* infection in children (grey circles) and adults (black circles) [outlier of 99,804 epg excluded].

Fig 4a and 4b depict the relationships between community-level hookworm infection indicators for both pre-school and school-aged children (2–14 years) and adults (≥15 years) separately for the 120 clusters. Correlation between prevalence of hookworm infection and arithmetic mean infection intensity were ρ = 0.75, p<0.001 and ρ = 0.71, p<0.001 for adults and children respectively (Fig 4a). A higher proportion of the MHI infections were found in adults (ρ = 0.77, p<0.001) (Fig 4b), consistent with patterns shown in Fig 3a. Whereas the relationship between prevalence of infection and MHI infection in school-aged children was substantially lower (ρ = 0.61, p<0.001), emphasising how the relationship between these two indicators may be perturbed after treatment (Fig 4b). In fact, in 23 clusters, prevalence of MHI infection exceeded the 1% threshold, despite the overall infection prevalence in these clusters lying below 20%. In contrast for *T*. *trichiura* the vast majority of MHI infections were found in children. The correlations between both prevalence and prevalence of MHI infections and the arithmetic mean infection intensity was much greater in children (ρ = 0.77, p<0.001) than adults (ρ = 0.42, p<0.001) (Fig 4c and 4d). Child-level prevalence of MHI infection exceeded the 1% threshold where overall infection prevalence was below 20% in 10 clusters.

### Correlates of hookworm infection presence and intensity

The majority of individual, household and community-level factors investigated were marginally associated with hookworm infection in the univariable analysis (S1 Table). Adjusting for other associated factors, many of these associations remained, with males and older age groups exhibiting greater odds of infection (Table 1). Age was found to significantly modify the association of sex with hookworm infection (p<0.0001). The difference in odds of infection between males and females was more pronounced across increasing age groups. Wearing shoes (Adjusted odds ratio [AOR] 0.71 (95% CI: 0.64–0.78), attending school and being dewormed in the last 12 months were all found to be protective behaviours for hookworm infection. Individuals from the least poor households had reduced odds of hookworm infection, whilst those in the poorest were at increased risk (Table 1). Several household WASH factors were also associated with reduced odds of infection, including access to a private latrine (AOR: 0.80 (95% CI: 0.68–0.94) and to an improved water source, as classified according to the WHO/UNICEF Joint Monitoring Programme for Water Supply, Sanitation and Hygiene (JMP) indicators [32]. A covered (man-made) floor halved the odds of infection compared to an earthen one. The influence of the community environment was observed, with positive associations between hookworm infection and high vegetation coverage, and humid conditions.

**Table 1:** Multivariable associations between hookworm (prevalence and intensity) and individual- household- and environmental factors across 120 clusters on the south coast of Kenya, 2015.

		Infected with hookworm/ total N (%)	Multivariable OR (95% CI) for hookworm infection†§	P value	Mean hookworm epg (SD)	Multivariable IRR (95% CI) for hookworm intensity‡^	P value
**INDIVIDUAL factors**							
**Sex**							
Male		1760 / 7871 (22.4)			211 (1309)	1	
Female		1191 / 11812 (16.9)			127 (901)	0.68 (0.53–0.86)	0.002
**Age**							
<5 years		196 / 1569 (12.5)			90 (720)	1	
5–14 years		1057 / 6066 (17.4)			93 (610)	0.73 (0.44–1.20)	
≥15 years		2497 / 12047 (20.7)			203 (1288)	1.44 (0.85–2.43)	<0.0001
**Effect of age by sex**							
Male:	<5 years	108 / 827 (13.1)	1				
	5–14 years	561 / 2954 (19.0)	2.47 (1.82–3.35)				
	≥15 years	1091 / 4090 (26.7)	3.47 (2.60–4.64)				
Female:	<5 years	88 / 742 (11.9)	1	<0.0001			
	5–14 years	496 / 3112 (15.9)	2.14 (1.57–2.93)				
	≥15 years	1406 / 7957 (17.7)	1.97 (1.43–2.72)				
**Effect of sex by age**							
<5 years:	Male	108 / 827 (13.1)	1				
	Female	88 / 742 (11.9)	0.93 (0.59–1.47)				
5–14 years:	Male	561 / 2954 (19.0)	1	<0.0001			
	Female	496 / 3112 (15.9)	0.81 (0.70–0.93)				
≥15 years:	Male	1091 / 4090 (26.7)	1				
	Female	1406 / 7957 (17.7)	0.53 (0.48–0.58)				
**Attend school**							
No		2561 / 12142 (21.1)	1				
Yes		1189 / 7540 (15.8)	0.80 (0.70–0.92)	0.001			
**Observed shoe type**							
No Shoes		2325 / 10824 (21.5)	1		181 (1045)	1	
Shoes		1422 / 8842 (16.1)	0.74 (0.67–0.80)	<0.0001	136 (1129)	0.70 (0.58–0.85)	0.001
**Received ALB (last 12months)**							
No		3101 / 14566 (21.3)	1		190 (1160)	1	
Yes		627 / 4921 (12.7)	0.67 (0.61–0.75)	<0.0001	78 (835)	0.63 (0.45–0.87)	0.003
**Open defecation**							
Yes		3101 / 14566 (21.3)	1				
No		627 / 4921 (12.7)	0.85 (0.74–0.99)	0.033			
**HOUSEHOLD factors**							
**Household SES**							
1 (Poorest)		1505 / 5997 (25.1)	1.19 (1.11–1.28)		246 (1349)	1.53 (1.19–1.96)	
2 (Middle)		1840 / 9888 (18.6)	1	< 0.0001	149 (1063)	1	<0.0001
3 (Least poor)		405 / 3797 (10.7)	0.69 (0.62–0.77)		56 (492)	0.42 (0.30–0.57)	
**Household flooring**							
Earth/sand		3350 / 15518 (21.6)	1				
Covered		400 / 4160 (9.6)	0.63 (0.55–0.71)	<0.0001			
**Reported toilet facility access**							
None		1988 / 9369 (21.2)	1				
Shared access		861 / 4613 (18.7)	0.87 (0.74–1.02)				
Private access		900 / 5691 (15.8)	0.80 (0.68–0.94)	0.023			
**Water Source**							
Non-Improved		2039 / 9219 (22.1)	1		193 (1193)	1	
Improved		1702 / 10406 (16.4)	0.88 (0.79–0.97)	0.011	133 (979)	0.74 (0.58–0.93)	0.012
**ENVIRONMENT factors**							
**Aridity**							
semi-arid		142 / 2355 (6.0)	1				
dry sub-humid		1237 / 6064 (20.4)	1.48 (0.90–2.46)				
Humid		2371 / 11263 (21.1)	2.66 (1.41–3.64)	<0.001			
**pH (KCl)**							
Low (<51)		1105 / 4774 (23.2)	1				
Medium (51–52)		1633 / 8210 (19.9)	0.76 (0.66–0.89)				
High (>52)		1012 / 6698 (15.1)	0.74 (0.62–0.90)	0.002			
**EVI**							
Low (<0.3)		625 / 6481 (9.6)	1		60 (491)	1	
Medium (0.3–0.4)		1511 / 6552 (23.1)	1.39 (1.20–1.60)		218 (1342)	2.75 (1.87–4.05)	
High (>0.4)		1614 / 6649 (24.3)	1.50 (1.28–1.77)	<0.0001	202 (1204)	2.40 (1.63–3.51)	<0.0001

^†^ Generalized estimating equations (GEE) with exchangeable correlation structure and logit link applied

^‡^Adjusted zero-inflated negative binomial regression model, inflating for sex, age (2-4years, 5–14 years, ≥15 years) and aridity, with a clustered sandwich estimator applied

^§^19,392 observations included in fully adjusted GEE model

^19,418 observations included in fully adjusted zero-inflated negative binomial regression model. All variables have complete data (with the exception of shoe type n = 19,666; received ALB n = 19,487; open defecation n = 19,625; flooring n = 19,678; latrine access n = 19,673 and water source n = 19,625). All data available displayed for “Infected with hookworm/ total N (%)” and “Mean hookworm epg (SD)”. Acronyms: albendazole (ALB), confidence interval (CI), environmental vulnerability index (EVI), incidence rate ratio (IRR), odds ratio (OR), potassium chloride (KCL), socioeconomic status (SES), standard deviation (SD)

There was a strong association between hookworm infection intensity and socio-economic status, with those in the poorest households having the heaviest infections and those in the highest wealth quintile the lightest (Table 1). Improved water sources were also related to lighter infections. Similarly, as with presence of infection, wearing shoes and being dewormed in the last year were protective against heavier infections, whereas being male and older in age were associated with heavier infections. In relation to environmental factors, only high levels of vegetation was associated with infection intensity.

### Correlates of *T*. *trichiura* infection presence and intensity

Several individual, household and environmental factors were associated with the presence of *T*. *trichiura* infection at the univariable level (S2 Table). After adjusting for other associated characteristics, only a few risk factors for infection remained. Males exhibited significantly higher odds of infection than females, and school-age children had substantially greater odds of infection than either preschool-age children or adults (Table 2). No evidence of an age-sex interaction was observed. Individuals in the poorest and least poor SES quintiles had increased and reduced odds of infection, respectively, when compared with the central three quintiles. Private latrine access was protective against both presence and intensity of infection. An arid environment and higher elevation were found to be protective against *T*. *trichiura* infection. A seven-fold increase in odds of infection was observed in more humid environments (Table 2) as these infections were almost exclusively restricted to the coast (Fig 2c). Similarly, low-lying locations were associated with substantially higher intensity *T*. *trichiura* infections. While attending school was associated with the higher intensity infections, having been treated in the last 12 months was associated with lower intensity infections.

**Table 2:** Multivariable associations between *T*. *trichiura* (prevalence and intensity) and individual- household- and environmental factors across 120 clusters on the south coast of Kenya, 2015.

	Infected with *T*. *trichiura* / total N (%)	Multivariable OR (95% CI) for *T*.*trichiura* infection†§	P value	Mean *T*.*trichiura* epg (SD)	Multivariable IRR (95% CI) for *T*.*trichiura* intensity‡^	P value
**INDIVIDUAL factors**						
**Sex**						
Male	336 / 7860 (4.3)	1				
Female	374 / 11806 (3.2)	0.79 (0.66–0.93)	0.004			
**Age**						
<5 years	55 / 1569 (3.5)	1				
5–14 years	314 / 6062 (5.2)	1.62 (1.15–2.28)				
≥15 years	341 / 12035 (2.8)	0.80 (0.56–1.14)	<0.0001			
**Attend school**						
No				7 (157)	1	
Yes				20 (292)	2.08 (1.26–3.43)	0.004
**Received ALB (last 12months)**						
No				11 (204)	1	
Yes				15 (262)	0.60 (0.38–0.97)	0.036
**HOUSEHOLD factors**						
**Household SES**						
1 (Poorest)	212 / 5990 (3.5)	1.14 (0.95–1.38)				
2 (Middle)	385 / 9880 (3.9)	1				
3 (Least poor)	113 / 3796 (3.0)	0.77 (0.60–0.99)	0.005			
**Reported toilet facility access**						
None	327 / 9361 (3.5)	1		12 (216)	1	
Shared access	205 / 4610 (4.5)	1.03 (0.85–1.25)		16 (266)	1.49 (0.76–2.92)	
Private access	178 / 5686 (3.1)	0.80 (0.65–0.98)	0.041	8 (179)	0.59 (0.35–0.97)	0.012
**ENVIRONMENT factors**						
**Altitude (metres)**						
Low (<59)	550 / 6501 (8.5)	1		33 (376)	1	
Medium (59–170)	120 / 6667 (1.8)	0.42 (0.28–0.61)		2 (34)	0.10 (0.05–0.20)	
High (>170)	40 / 6498 (0.6)	0.26 (0.16–0.42)	<0.0001	1 (48)	0.10 (0.03–0.29)	<0.0001
**Aridity**						
Semi-arid	7 / 2355 (0.3)	1				
Dry sub-humid	58 / 6060 (1.0)	2.60 (1.09–6.18)				
Humid	645 / 11251 (5.7)	8.06 (3.39–19.15)	<0.0001			

^†^ Generalized estimating equations with exchangeable correlation structure and logit link applied

^‡^Adjusted zero-inflated negative binomial regression model, inflating for sex, age (2–4 years, 5–14 years, ≥15 years) and aridity, with a clustered sandwich estimator applied

^§^19,657 observations included in fully adjusted GEE model

^19,462 observations included in fully adjusted zero-inflated negative binomial regression model. All variables have complete data (with the exception of attend school n = 19,666; received ALB n = 19,471; latrine access n = 19,657). All data available displayed for “Infected with *T*. *trichiura*/ total N (%)” and “Mean *T*. *trichiura* hookworm epg (SD)” Acronyms: albendazole (ALB), confidence interval (CI), incidence rate ratio (IRR), odds ratio (OR), socioeconomic status (SES), standard deviation (SD)

### The relationship between STH and sanitation

To explore further the relationship between STH and sanitation, we conducted a sub-analysis of toilet facility type, construction, and cleanliness among households that reported access to a toilet facility and permitted us to conduct an observation of the sanitation facilities (n = 7791). After adjusting for socio-economic status, access to a latrine constructed with man-made materials and/or a washable slab was associated with reduced odds of hookworm infection. Whilst the latrine type did not appear to be associated with the odds of hookworm infection, cleanliness did, as those with visible faeces round the edge of the latrine, had a 30% increased odds of infection (Table 3). Access to a handwashing station with water and soap was protective, with a 30% reduction in odds of hookworm infection, compared with no handwashing station or one with only water available. In contrast, sanitation conditions and handwashing facilities in those households with access did not appear to be associated with the presence of *T*. *trichiura* infection.

**Table 3:** Associations between presence of hookworm and *T*. *trichiura* infection with sanitation characteristics and conditions, adjusting for household socioeconomic status (N = 7791).

Observed Access N = 7791*	N	Hook worm n	Hook worm %	OR†‡ hookworm infection (95% CI)	p-value	*T*. *trichiura* n	*T*. *trichiura* %	OR†‡ *T*. *trichiura* infection (95% CI)	p-value
**Type of toilet facility**									
Pit latrine	6976	1160	16.63	1		214	3.07	1	
Ventilated improved pit latrine	457	46	10.07	0.86 (0.66–1.13)		13	2.85	0.62 (0.33–1.15)	
Water-borne	358	37	10.34	0.90 (0.66–1.25)	0.457	17	4.75	1.00 (0.54–1.85)	0.318
**Visible faeces around the edge**									
No	7010	1065	15.19	1		227	3.24	1	
Yes	781	178	22.79	1.30 (1.08–1.57)	0.006	17	2.18	0.87 (0.62–1.23)	0.482
**Washable slab**									
No (natural material e.g. timber)	3741	833	22.27	1		81	2.17	1	
Yes (tile or cement)	4050	410	10.12	0.67 (0.58–0.77)	<0.0001	163	4.03	1.10 (0.89–1.36)	0.364
**Toilet facility walls**									
No walls	218	59	27.06	1		5	2.29	1	
Natural material	4140	882	21.30	0.69 (0.51–0.94)		106	2.56	1.23 (0.61–2.46)	
Man made material	3433	302	8.80	0.42 (0.30–0.58)	<0.0001	133	3.87	1.15 (0.58–2.27)	0.770
**Toilet facility roof**									
No roof	1253	228	18.20	1		55	4.39	1	
Natural material	2971	678	22.82	1.02 (0.84–1.24)		80	2.70	0.94 (0.67–1.32)	
Man made material	3567	337	9.45	0.61 (0.52–0.72)	<0.0001	109	3.06	0.82 (0.57–1.20)	0.484
**Hand washing facilities**†									
None designated or no water	6740	1115	16.54	1		208	3.09	1	
Yes, only water	367	61	16.62	1.20 (0.92–1.56)		6	1.63	0.46 (0.20–1.05)	
Yes, water and soap	567	42	7.41	0.69 (0.52–0.92)	0.001	28	4.94	1.19 (0.75–1.33)	0.118

* Including only those with access to a toilet facility with directly observed sanitation facilities

^†^117 missing observations for handwashing facilities variable. All other variables have 7791 observations.

^‡^Analyses adjust for SES only

## Discussion

This study of almost 20,000 individuals in coastal Kenya provides a detailed analysis of the community-level epidemiology of STH within the context of ongoing school-based treatment. Given the rapid uptake of national school-based deworming programmes across much of the continent, findings presented here are likely to be broadly comparable to large parts of SSA where the STH epidemiological and demographic profiles are similar.

The observed hookworm prevalence of 19.1% was lower than the prevalence of 42% observed in a convenience sample of adults in the same coastal region of Kenya in 2010 [17] but was in line with a community-wide parasite survey conducted in four sentinel villages in the region between 2009 and 2011, which suggested a hookworm prevalence of 21% [18]. The NSBDP reported a substantially lower prevalence of 9.7%, 2.4% and 0.4% for hookworm, *T*. *trichiura* and *A*. *lumbricoides* respectively in school children in Kwale in February 2015 [33]. The increase in hookworm prevalence and intensity with age across both sexes, although higher in males, is a pattern similar to that observed in the majority of published age-structured cross sectional surveys [6, 12, 34]. Taken together, these observations suggest that despite notable declines in STH infection in school-going children following three years of school-based deworming, the programme appears to have had less of an impact on prevalence of hookworm in adults and non-school-going children. More recently, results of a national evaluation in 2017, following five years of school-based deworming in Kenya, indicate that despite substantial reductions in STH prevalence, complementary interventions may be required in some settings moving forward [35]. This conclusion is in line with the epidemiology of hookworm and modelling results, which show that in most settings school-based deworming alone is insufficient to reduce hookworm transmission within communities [6, 36].

The WHO-stated aim is to reduce STH infection in school-age children to less than 1% MHI infections, under the assumption that STH-related morbidity is no longer a public health problem at this level [23]. The results presented here demonstrate that after three consecutive years of school-based deworming, Kwale sits just above this 1% threshold, with substantial heterogeneity between clusters. However, the sizeable burden of MHI infections is in adults (2.4%, males 3.4% and females 1.9%), who remain vulnerable to the anaemia associated with harbouring heavy hookworm burdens, especially women of childbearing age [34, 37]. This suggests that targeting adults for deworming may have important consequences for both morbidity and transmission control in areas where STH transmission is dominated by hookworm. Conversely, our results suggest that children remain the most important targets for control and surveillance when *T*. *trichiura* is the dominant species.

Understanding the nuanced relationship between prevalence and intensity of infection can be helpful when evaluating the relevance of these indicators for monitoring and surveillance of control programmes [1]. It is widely recognised that measures of infection intensity are better indicators of transmission and public health impact in communities than prevalence alone [38–40]. Control measures such as repeated MDA tend to alter the pattern of aggregation of the parasites in communities (as measured by the negative binomial parameter k) and concomitantly significantly alter the relationship between prevalence and intensity [41]. For low values, the prevalence is roughly constant across a wide range of mean intensity values. Prevalence in such circumstances is a poor predictor of the number of high intensity infections. The weak associations between prevalence and intensity observed here, especially for hookworm in school-aged populations, are a reflection of this pattern.

This study emphasises the large degree of heterogeneity in STH infection risk across a relatively small geographic area, and confirms a number of well-known risk factors for STH infection such as the protective effect of shoe wearing in relation to hookworm. In addition to highlighting established environmental risk factors which are known to affect suitability for transmission including elevation, aridity, vegetation and soil pH [42, 43], we demonstrate how the relationship between infection and poverty is enduring across species, even after accounting for associated proximal risk factors such as sanitation. This finding further underlines how the poorest in endemic communities continue to experience the highest infection burden after years of control, emphasising the importance of developing equitable strategies that reach the very poorest community members. The poorest people in communities may also have low adherence to repeated rounds of deworming and low coverage at each round. For example, 78% of the children included in our survey reported attending school, although this drops from 86% in the highest quintile to 72% in the poorest quintile whom also experience the greatest infection risk. New strategies are therefore required to reach non-school-attending children in a sustainable and equitable manner. It would seem most feasible to target these individuals alongside the wider population through a community-wide treatment approach, as currently employed by the lymphatic filariasis and onchocerciasis elimination programmes. It would be essential to try and ensure high MDA coverage and good compliance over repeated rounds of MDA in such individuals.

Latrine access has commonly been reported as associated with reduced risk of STH infection [44–46], and as can be seen from these results, access to a toilet facility was associated with reduced odds of both hookworm and *Trichuris* infection. However, our evidence suggests that only access to a private toilet is associated with reduced infection and that shared access confers no benefits in preventing infection. This finding lends support to the guidelines that shared sanitation access be considered as basic sanitation, with private household access as improved sanitation [47, 48]. Shared latrines are argued to be unimproved for reasons of reduced quality and cleanliness, and the finding in this setting of decreased hookworm infection in individuals with better constructed and cleaner latrines lends support to the theory that poorly constructed and poorly maintained sanitation facilities can be harmful [46, 49]. Poorly maintained toilet facilities may concentrate faeces in an environment that individuals repeatedly come into contact with, thereby increasing harmful exposure. Similar associations between *A*. *lumbricoides* and latrine structure and cleanliness have been found in surveys of school WASH in Kenya [46].

The highly focal distribution of *T*. *trichiura* infection concentrated in the communities along the coast, although consistent with previous findings, warrants further investigation [50]. It is widely accepted that the current benzamidazoles (albendazole and mebendazole) demonstrate limited efficacy against *T*. *trichiura*, with reported cure rates as low as 28% [51, 52]. This reduced efficacy would appear to be supported by our findings, as there is a notable absence of any reduction in infection risk in those individuals reporting dewormed in the last 12 months, in direct contrast to hookworm, although deworming was found associated with reduced *T*. *trichiura* intensity. However, treatment efficacy does not fully explain the highly clustered nature of this infection. Possible explanations could relate to uncaptured environmental characteristics, unmeasured behavioural or hygiene-related practices along the shoreline, or a localised history of poor treatment coverage. Interestingly, we observed an absence of association between *T*. *trichiura* infection and handwashing and toilet facility conditions in those with access, which is perhaps surprising but suggests that other hygiene-related factors may be more influential in this setting.

Despite the unique scope of this robust survey, there are several important limitations. First, we encountered challenges inherent in community-based stool sampling that may have led to some bias in our final sample. We recruited participants in their homes by randomly selecting from all listed household members. Selected members whom we could not find at home or at a nearby location were replaced by another randomly selected household member. This replacement approach may have contributed to the gender imbalance observed in our sampled population. Such imbalances are frequently encountered in community-based surveys, as women and young children are more likely to be found at home during the day, while the population engaged in activities outside the home is absent. [18, 53]. The use of a population census sampling frame—as opposed to the household census sampling frame used here—could have potentially reduced any bias introduced by on-the-spot randomisation and limited replacement from the selected households.

Additionally, Kato-Katz thick smear method (a commonly used field assessment tool for detection of STH infection presence and intensity) is known to have low sensitivity at low levels of infection [33, 41, 54, 55]. Consecutive day sampling for assessment with Kato-Katz would likely have increased the diagnostic accuracy, with evidence from western Kenya indicating greater than 20% increase in sensitivity of Kato-Katz when assessing consecutive samples [56]. However, duplicate stool collection was not logistically feasible for such a large sample in this study.

An improved understanding of the distribution of STH infections and factors associated with increased risk in endemic communities is vital if we are to fully understand the impact of current and proposed STH control and elimination strategies. The data reported here show that even after repeated rounds of school-based deworming, hookworm remains prevalent throughout Kwale County, with highest prevalence and intensity observed in adult men. This suggests that school-based deworming has been insufficient to control community-wide hookworm infection, and we instead need to evaluate the impact and cost-effectiveness of community-wide treatment strategies. Our results also confirm that to have greatest impact, treatment strategies not only need to reach a wider age-range, but also those most vulnerable to infection, including the very poorest households and those without access to sanitation. An increased focus on ensuring high coverage and good compliance in the poorest families must be a priority in future efforts to control STH infection.

## Supporting information

S1 Text Environmental factors. (PDF)

S1 Fig Geographic distribution of (A) soil pH [potassium chloride KCL] (B) sand fraction in soil and (C) urbanization in Kwale county, south coast of Kenya, 2015. Also shown is the location of Kwale County in Kenya.
(PDF)

S2 Fig Geographic distribution of (A) enhanced vegetation index (EVI) (B) land surface temperature (LST) (C) aridity and (D) elevation (m) in Kwale county, south coast of Kenya, 2015. (PDF)

S1 Table Univariable associations between both hookworm prevalence and intensity and individual- household- and environmental factors across 120 clusters on the south coast of Kenya, 2015. (PDF)

S2 Table Univariable associations between both *T*. *trichiura* prevalence and intensity and individual- household- and environmental factors across 120 clusters on the south coast of Kenya, 2015. (PDF)

S3 Table Individual- household- and environmental factors for individuals with matched parasitological data vs. those individuals without matched parasitological data across 120 clusters on the south coast of Kenya, 2015. (PDF)

S1 Checklist STROBE checklist. (PDF)
