# Supplementary material for: Community-level epidemiology of soil-transmitted helminths in the context of school-based deworming: Baseline results of a cluster randomised trial on the coast of Kenya
Source: PLoS Negl Trop Dis. 2019 Aug 9;13(8):e0007427. doi: 10.1371/journal.pntd.0007427 (PMC6719894; doi:10.1371/journal.pntd.0007427)
Supplement: S1 Text — (PDF) [file pntd.0007427.s001.pdf]

S1 Text.

Environmental indicators

A suite of environmental and topographic datasets were explored as potential environmental drivers of STH in the study area. Vegetation coverage and land surface temperature were obtained by processing moderate resolution satellite images provided by the Moderate Resolution Imaging Spectroradiometer (MODIS) instrument operating in the Terra spacecraft (NASA), which measure 36 spectral bands and it acquires data at lowest spatial resolution of 250m. From the family of MODIS products, we downloaded fortnightly MOD13Q1 [1] and MOD11A2 [2] data, which are intended to monitor vegetation coverage and land surface temperature respectively. These MODIS datasets were acquired for the period three months before the baseline survey. Fortnightly continuous gridded maps of Enhanced Vegetation Index (EVI) and Land Surface Temperature (LST) were produced and eventually aggregated by calculating the mean for the period.

From the Consortium for Spatial Information (CGIAR-CSI), we obtained a raster dataset of elevation at 1km^2 [3]. This elevation layer resulted from processing and resampling the gridded digital elevation models (DEM) derived from the original 30-arcsecond DEM produced by the Shuttle Radar Topography Mission (SRTM). An aridity index, which is a generalized function of precipitation, temperature, and/or potential evapotranspiration, was obtained at 1 km resolution from CGIAR-CSI [3]. Estimates of soil acidity (pH KCL) and sand content were extracted from soilgrids.org at a resolution of 250m [4]. Elevation, aridity index and soil-related datasets are not subjected to a specific period of time, and we used the most updated versions produced by Shuttle Radar Topography Mission (SRTM) for elevation, CGIAR-CSI for aridity index, and World Soil project for pH KCl.

Population density estimates have been produced by WorldPop project, which provide country level gridded maps of population density for different years (i.e 2000, 2010, 2015) and projections (2020) [5]. Estimates of population density for 2015 (the population estimate nearest in time to the study period) were obtained from the WorldPop project, which was used to classify areas as urban, peri-urban or rural areas, based on the assumption that urban extents (UE) have a population densities ≥1,000 persons/km^2, peri-urban >250 persons/km^2 within a 15 km distance from UE edge, and rural <250 persons/km^2 and/or >15 km from the UE edge [6]. The range of environmental and topographic data were extracted using ArcGIS 10.3 (Environmental Systems Research Institute Inc. Redlands, CA, US). The range of environmental and topographic data were extracted using point-based extraction for each household. Households without GPS coordinates were given the village mean or mode value for continuous and categorical environmental measures, respectively.

1. DAAC, N.L., *NASA LP DAAC: MOD13Q1 Vegetation Indices 16-Day L3 Global 250m*, NASA EOSDIS Land Processes DAAC, USGS Earth Resources Observation and Science (EROS) Center, Sioux Falls, South Dakota (https://lpdaac.usgs.gov).
2. DAAC, N.L., *NASA LP DAAC: MOD11A2 Land Surface Temperature and Emissivity 8-Day L3 Global 1km*, NASA EOSDIS Land Processes DAAC, USGS Earth Resources Observation and Science (EROS) Center, Sioux Falls, South Dakota (https://lpdaac.usgs.gov).
3. CGIAR-CSI. *Consortium for Spatial Information*. Available from: http://www.cgiar-csi.org/.
4. Hengl, T., et al., *SoilGrids250m: Global gridded soil information based on machine learning.* PLoS one, 2017. **12**(2): p. e0169748.
5. WorldPop. *The WorldPop demography project*. Available from: http://www.worldpop.org.uk/.
6. Pullan, R.L. and S.J. Brooker, *The global limits and population at risk of soil-transmitted helminth infections in 2010.* Parasit Vectors, 2012. **5**: p. 81.
