# Supplementary material for: Community-level epidemiology of soil-transmitted helminths in the context of school-based deworming: Baseline results of a cluster randomised trial on the coast of Kenya
Source: PLoS Negl Trop Dis. 2019 Aug 9;13(8):e0007427. doi: 10.1371/journal.pntd.0007427 (PMC6719894; doi:10.1371/journal.pntd.0007427)

S1 Figure: Geographic distribution of (A) soil pH [potassium chloride KCL] (B) sand fraction in soil and (C) urbanization in Kwale county, south coast of Kenya, 2015. Also shown is the location of Kwale County in Kenya.

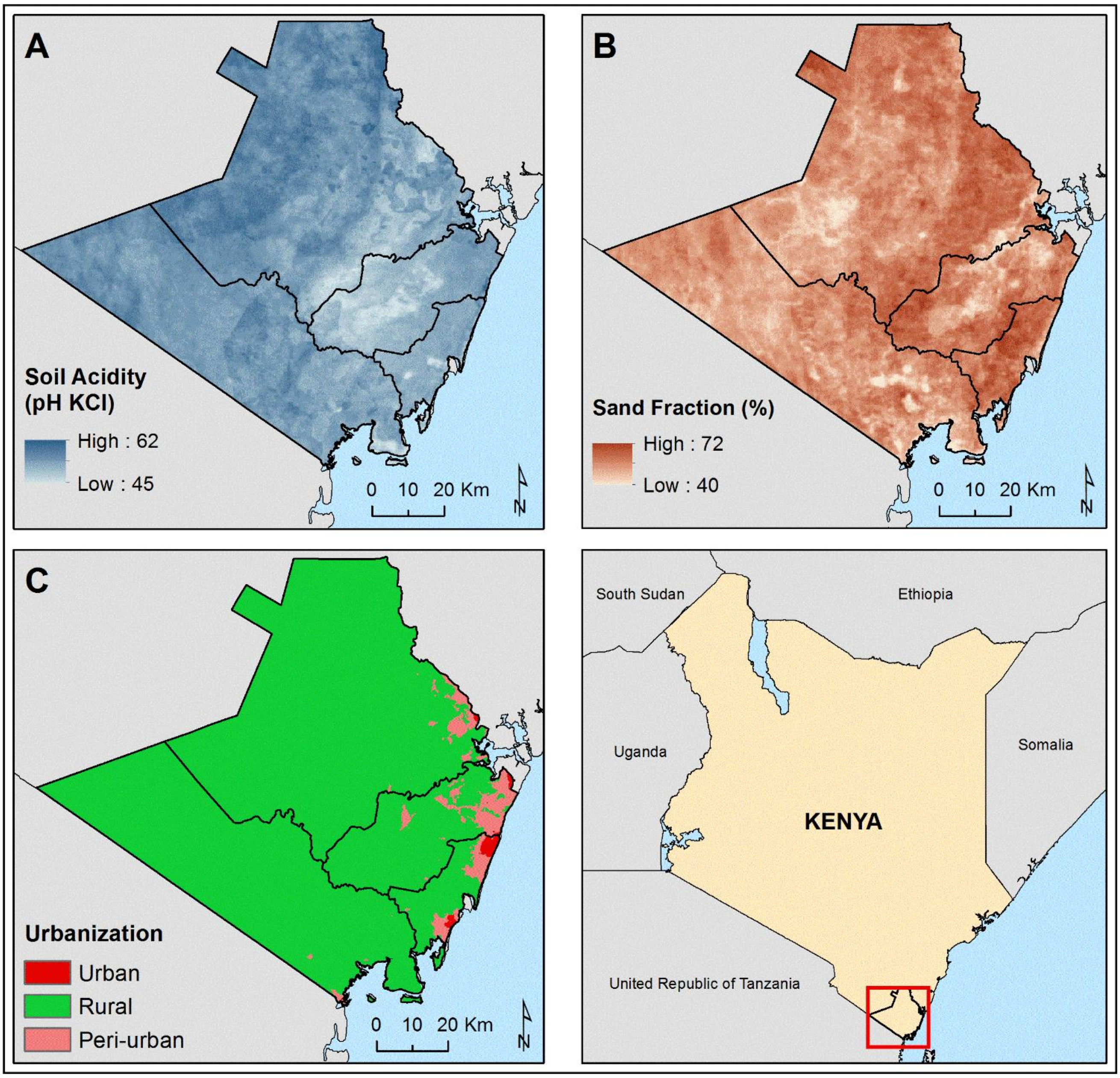

Supplement: S1 Fig — Also shown is the location of Kwale County in Kenya. (PDF) [file pntd.0007427.s002.pdf]
