# Supplementary material for: Community-level epidemiology of soil-transmitted helminths in the context of school-based deworming: Baseline results of a cluster randomised trial on the coast of Kenya
Source: PLoS Negl Trop Dis. 2019 Aug 9;13(8):e0007427. doi: 10.1371/journal.pntd.0007427 (PMC6719894; doi:10.1371/journal.pntd.0007427)

S2 Figure. Geographic distribution of (A) enhanced vegetation index (EVI) (B) land surface temperature (LST) (C) aridity and (D) elevation (m) in Kwale county, south coast of Kenya, 2015.

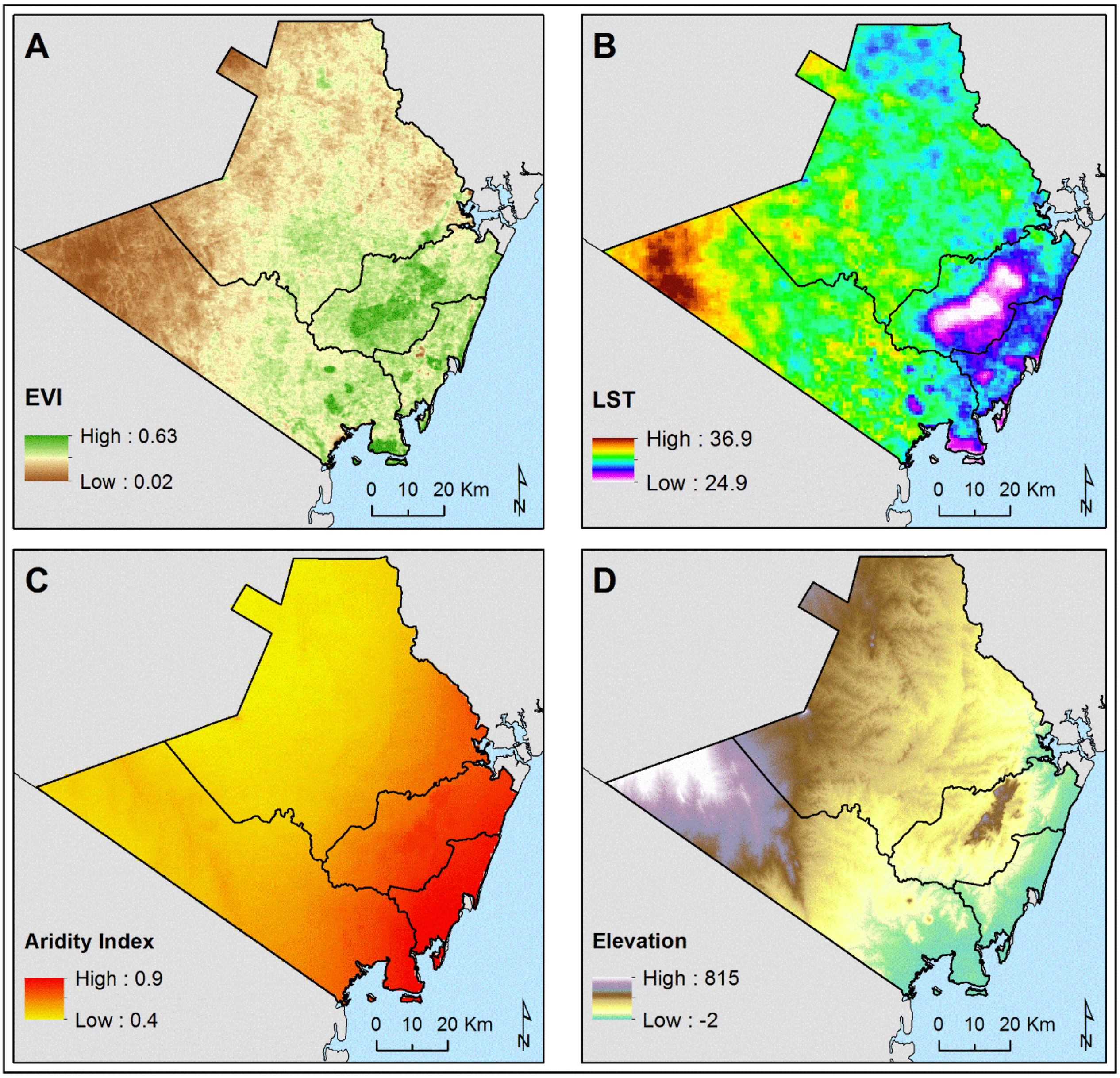

Supplement: S2 Fig — (PDF) [file pntd.0007427.s003.pdf]
