# Supplementary material for: Community-level epidemiology of soil-transmitted helminths in the context of school-based deworming: Baseline results of a cluster randomised trial on the coast of Kenya
Source: PLoS Negl Trop Dis. 2019 Aug 9;13(8):e0007427. doi: 10.1371/journal.pntd.0007427 (PMC6719894; doi:10.1371/journal.pntd.0007427)
Supplement: S1 Table — (PDF) [file pntd.0007427.s004.pdf]

S1 Table. Univariable associations between both hookworm prevalence and intensity and individual- household- and environmental factors across 120 clusters on the south coast of Kenya, 2015.

	Number* ˣ (N) [%]	Number infected (n) [%]	Univariable OR (95% CI) for hk infection†	P value	Mean epg [SD]	Univariable IRR (95% CI) for hk intensity‡	P value
**INDIVIDUAL FACTORS**							
**Sex**							
Male	7871 (40.0)	1760 (22.4)	1		211 (1309)	1	
Female	11811 (60.0)	1990 (16.9)	0.72 (0.66-0.77)	<0.0001	127 (901)	0.77 (0.63-0.94)	0.009
**Age**							
<5 years	1569 (8.0)	196 (12.5)	1		90 (720)	1	
5-14 years	6066 (30.8)	1057 (17.4)	1.64 (1.39-1.94)		93 (610)	0.69 (0.47-1.02)	<0.0001
≥15 years	12047 (61.2)	2497 (20.7)	2.05 (1.73-2.42)	<0.0001	203 (1288)	1.42 (0.92-2.19)	
**Attend school**							
No	12142 (61.7)	2561 (21.1)	1		206 (1237)	1	
Yes	7540 (38.3)	1189 (15.8)	0.73 (0.66-0.81)	<0.0001	88 (769)	0.46 (0.36-0.58)	<0.0001
**Received ALB (last 12months)**							
No	14566 (74.7)	3101 (21.3)	1		190 (1160)	1	
Yes	4921 (25.3)	627 (12.7)	0.59 (0.53-0.65)	<0.0001	78 (835)	0.47 (0.34-0.64)	<0.0001
**Observed shoe type**							
No Shoes	10824 (55.0)	2325 (21.5)	1		181 (1045)	1	
Shoes	8842 (45.0)	1422 (16.1)	0.76 (0.70-0.83)	<0.0001	136 (1129)	0.76 (0.61-0.93)	0.008
**Open defecation§**							
Yes	10586 (53.9)	2229 (21.1)	1		175 (1078)	1	
No	9053 (46.1)	1514 (16.7)	0.63 (0.57-0.70)	<0.0001	144 (1092)	0.79 (0.62-1.01)	0.059
**HOUSEHOLD FACTORS**							
**Household SES**							
1 (Poorest)	5997 (30.5)	1505 (25.1)	1.36 (0.28-1.45)		246 (1349)	1.54 (1.25-1.88)	
2 (Middle)	9888 (50.2)	1840 (18.6)	1		149 (1063)	1	<0.0001
3 (Least poor)	3797 (19.3)	405 (10.7)	0.56 (0.51-0.61)	<0.0001	56 (492)	0.39 (0.29-0.54)	
**Household flooring**							
Earth/sand	15518 (78.9)	3350 (21.6)	1		183 (1124)	1	
Covered	4160 (21.1)	400 (9.6)	0.44 (0.39-0.50)	<0.0001	75 (910)	0.43 (0.27-0.68)	<0.001
**Reported toilet facility access**							
None	9369 (47.6)	1988 (21.2)	1		179 (1104)	1	
Shared access	4613 (23.5)	861 (18.7)	0.67 (0.60-0.75)		169 (1209)	0.91 (0.68-1.20)	0.014
Private access	5691 (28.9)	900 (15.8)	0.54 (0.48-0.62)	<0.0001	124 (930)	0.66 (0.50-0.88)	
**Water Source**							
Non-Improved	9219 (47.0)	2039 (22.1)	1		193 (1193)	1	
Improved	10406 (53.0)	1702 (16.4)	0.77 (0.70-0.85)	<0.0001	133 (978)	0.70 (0.57-0.87)	0.001
**≤ 30 mins to water source**							
No	3812 (19.5)	773 (20.3)	1		187 (1101)	1	
Yes	15769 (80.5)	2962 (18.8)	0.97 (0.87-1.07)	0.531	154 (1082)	0.81 (0.61-1.07)	0.143
**ENVIRONMENT FACTORS**							
**Urban/ rural**							
Rural	14682 (74.6)	2989 (20.4)	1		170 (1066)	1	
Periurban	3817 (19.4)	625 (16.4)	0.92 (0.75-1.13)		146 (1230)	0.80 (0.56-1.16)	
Urban	1183 (6.0)	136 (11.5)	0.47 (0.29-0.74)	<0.001	93 (725)	0.54 (0.30-1.00)	0.085
**Aridity**							
Semi-arid	2355 (12.0)	142 (6.0)	1		28 (295)	1	
Dry sub-humid	6064 (30.8)	1237 (20.4)	1.27 (0.77-2.09)	0.029	165 (1041)	1.92 (1.33-2.78)	<0.0001
Humid	11263 (57.2)	2371 (21.1)	1.73 (1.06-2.81)		186 (1202)	2.12 (1.54-2.91)	
**Altitude (metres)**							
Low (<59)	6510 (33.1)	1234 (19.0)	1		180 (1241)	1	
Medium (59-170)	6674 (33.9)	1569 (23.5)	1.18 (0.94-1.48)	0.350	196 (1106)	1.04 (0.79-1.37)	0.269
High (>170)	6498 (33.0)	947 (14.6)	0.15 (0.86-1.53)		105 (867)	0.79 (0.55-1.12)	
**EVI**							
Low (<0.3)	6481 (32.9)	625 (9.6)	1		60 (491)	1	
Medium (0.3-0.4)	6552 (33.3)	1511 (23.1)	1.39 (1.21-1.60)	<0.0001	219 (1342)	2.66 (1.86-3.82)	<0.0001
High (>0.4)	6649 (33.8)	1614 (24.3)	1.52 (1.29-1.80)		202 (1204)	2.34 (1.66-3.29)	
**Sand content of soil (%)**							
Low (<59)	4865 (24.7)	1007 (20.7)	1		147 (765)	1	
Medium (59-61)	8020 (40.7)	1358 (16.9)	1.00 (0.86-1.15)	0.316	138 (1029)	1.01 (0.78-1.31)	0.034
High (>61)	6797 (34.5)	1385 (20.4)	1.09 (0.93-1.27)		197 (1315)	1.35 (1.06-1.71)	

**pH (KCl)**							
Low (<51)	4774 (24.3)	1105 (23.1)	1		193 (1280)	1	
Medium (51-52)	8210 (41.7)	1633 (19.9)	0.82 (0.70-0.96)	0.035	182 (1157)	1.01 (0.75-1.37)	0.069
High (>52)	6698 (34.0)	1012 (15.1)	0.80 (0.66-0.97)		111 (797)	0.74 (0.51-1.07)	

* A total of 16,682 individuals included with hookworm outcome data. The two outliers (one with hookworm intensity of 137,460epg and one with *T. trichiura* intensity of 99,804epg are excluded).

ˣ All characteristics have less than 1% missing data

† Generalised estimating equations (GEE) with exchangeable correlation structure and logit link applied for the relationships with prevalence

‡Zero-inflated negative binomial regression model, inflating for sex, age (2-4years, 5-14 years, ≥15 years) and aridity, with a clustered sandwich estimator applied for the relationships with intensity

§Open defecation refers to whether the individual used a latrine when they last defecated or if under 3 years whether their stool was disposed of safely.

Acronyms: albendazole (ALB), confidence interval (CI), environmental vulnerability index (EVI), incidence rate ratio (IRR), odds ratio (OR), potassium chloride (KCL), socioeconomic status (SES), standard deviation (SD)
