# Supplementary material for: Community-level epidemiology of soil-transmitted helminths in the context of school-based deworming: Baseline results of a cluster randomised trial on the coast of Kenya
Source: PLoS Negl Trop Dis. 2019 Aug 9;13(8):e0007427. doi: 10.1371/journal.pntd.0007427 (PMC6719894; doi:10.1371/journal.pntd.0007427)
Supplement: S2 Table — (PDF) [file pntd.0007427.s005.pdf]

S2 Table. Univariable associations between both *T. trichiura* prevalence and intensity and individual- household- and environmental factors across 120 clusters on the south coast of Kenya, 2015.

	Number (N) **	Number infected (n)	Univariable OR (95% CI) for *T. trichiura* infection†	P value	Mean epg (SD)	Univariable IRR (95% CI) for *T. trichiura* intensity‡	P value
**INDIVIDUAL FACTORS**							
**Sex**							
Male	7860 (40)	336 (4.3)	1		14 (223)	1	
Female	11806 (60)	374 (3.2)	0.74 (0.64-0.86)	<0.0001	10 (217)	0.95 (0.51-1.77)	0.873
**Age**							
<5 years	1569 (8.0)	55 (3.5)	1		33 (528)	1	
5-14 years	6062 (30.8)	314 (5.2)	1.53 (1.20-2.22)		18 (248)	0.31 (0.12-0.82)	<0.0001
≥15 years	12035 (61.2)	341 (2.8)	0.76 (0.56-1.04)	<0.0001	6 (105)	0.15 (0.06-0.40)	
**Attend school**							
No	12130 (61.7)	344 (2.8)	1		7 (157)	1	
Yes	7536 (38.3)	366 (4.9)	1.73 (1.44-2.08)	<0.0001	20 (292)	2.03 (1.12-3.68)	0.019
**Received ALB (last 12months)**							
No	14552 (74.7)	494 (3.4)	1		11 (204)	1	
Yes	4919 (25.3)	209 (4.3)	1.31 (1.13-1.54)	<0.001	15 (262)	1.13 (0.60-2.11)	0.713
**Observed shoe type**							
No Shoes	10813 (55.0)	358 (3.3)	1		12 (257)	1	
Shoes	8837 (45.0)	352 (4.0)	0.97 (0.83-1.12)	0.653	11 (162)	0.87 (0.52-1.45)	0.582
**Open defecation§**							
Yes	10577 (53.9)	379 (3.6)	1		12 (215)	1	
No	9046 (46.1)	331 (3.7)	0.77 (0.64-0.93)	0.008	11 (224)	0.65 (0.35-1.21)	0.174
**HOUSEHOLD FACTORS**							
**Household SES**							
1 (Poorest)	5990 (30.5)	212 (3.5)	1.13 (0.97-1.32)		11 (219)	0.84 (0.43-1.64)	
2 (Middle)	9880 (50.2)	385 (3.9)	1		14 (248)	1	0.049
3 (Least poor)	3796 (19.3)	113 (3.0)	0.73 (0.57-0.92)	<0.001	6 (116)	0.44 (0.23-0.85)	
**Household flooring**							
Earth/sand	15506 (78.9)	498 (3.2)	1		11 (215)	1	
Covered	4156 (21.1)	212 (5.1)	0.79 (0.60-1.04)	0.090	14 (235)	0.92 (0.51-1.65)	0.781
**Reported toilet facility access**							
None	9361 (47.6)	327 (3.5)	1		12 (216)	1	
Shared access	4610 (23.5)	205 (4.5)	0.99 (0.83-1.19)		15 (266)	0.87 (0.42-1.81)	0.058
Private access	5686 (28.9)	178 (3.1)	0.73 (0.59-0.91)	0.009	8 (179)	0.42 (0.21-0.87)	
**Water Source**							
Non-Improved	9210 (47.0)	189 (2.1)	1		7 (218)	1	
Improved	10399 (53.0)	517 (5.0)	1.26 (1.02-1.56)	0.035	16 (221)	1.36 (0.64-2.88)	0.427
**≤ 30 minutes to water source**							
No	3809 (19.5)	54 (1.4)	1		4 (121)	1	
Yes	15756 (80.5)	652 (4.1)	1.24 (1.04-1.47)	0.014	14 (238)	1.81 (0.71-4.59)	0.212
**ENVIRONMENT FACTORS**							
**Urban/ rural**							
Rural	14670 (74.6)	395 (2.7)	1		9 (179)	1	
Periurban	3817 (19.4)	218 (5.7)	1.56 (1.10-2.21)		15 (233)	0.97 (0.48-1.94)	
Urban	1179 (6.0)	97 (8.3)	1.31 (0.51-3.67)	0.043	41 (476)	2.51 (0.98-6.41)	0.113
**Aridity**							
Semi-arid	2355 (12.0)	7 (0.3)	1		0 (3)	1	
Dry sub-humid	6060 (30.8)	58 (1.0)	3.80 (1.62-8.90)		1 (10)	1.54 (0.47-5.03)	<0.0001
Humid	11251 (57.2)	645 (5.7)	13.44 (5.85-30.88)	<0.0001	20 (289)	16.37 (5.20-51.53)	
**Altitude (metres)**							
Low (<59)	6501 (33.1)	550 (8.5)	1		33 (376)	1	
Medium (59-170)	6667 (33.9)	120 (1.8)	0.43 (0.29-0.64)		2 (34)	0.09 (0.05-0.18)	<0.0001
High (>170)	6498 (33.0)	40 (0.6)	0.23 (0.15-0.35)	<0.0001	1 (48)	0.10 (0.03-0.36)	
**EVI**							
Low (<0.3)	6480 (32.9)	98 (1.5)	1		3 (74)	1	
Medium (0.3-0.4)	6543 (33.3)	284 (4.4)	1.28 (0.95-1.73)		16 (284)	2.89 (1.28-6.51)	0.035
High (>0.4)	6643 (33.8)	328 (4.9)	1.34 (0.95-1.88)	0.193	16 (240)	2.22 (1.04-4.74)	
**Sand content of soil (%)**							
Low (<59)	4862 (24.7)	193 (4.0)	1		14 (259)	1	
Medium (59-61)	8014 (40.7)	227 (2.8)	0.98 (0.77-1.27)		9 (185)	0.77 (0.35-1.69)	0.435
High (>61)	6790 (34.5)	290 (4.3)	1.23 (0.89-1.69)	0.179	14 (225)	1.23 (0.54-2.80)	

**pH (KCl)**							
Low (<51)	4771 (24.3)	128 (2.7)	1		12 (244)	1	
Medium (51-52)	8204 (41.7)	443 (5.4)	1.28 (0.84-1.95)		17 (241)	1.15 (0.44-3.02)	0.454
High (>52)	6691 (34.0)	139 (2.1)	1.07 (0.69-1.67)	0.309	5 (165)	0.67 (0.21-2.11)	

* A total of 16,666 individuals included with *T. trichiura* outcome data. The two outliers (one with hookworm intensity of 137,460epg and one with *T. trichiura* intensity of 99,804epg are excluded).

[x] All characteristics have less than 1% missing data

† Generalised estimating equations (GEE) with exchangeable correlation structure and logit link applied for the relationships with prevalence

‡Zero-inflated negative binomial regression model, inflating for sex, age (2-4years, 5-14 years, ≥15 years) and aridity, with a clustered sandwich estimator applied for the relationships with intensity

§Open defecation refers to whether the individual used a latrine when they last defecated or if under 3 years whether their stool was disposed of safely.

Acronyms: albendazole (ALB), confidence interval (CI), environmental vulnerability index (EVI), incidence rate ratio (IRR), odds ratio (OR), potassium chloride (KCL), socioeconomic status (SES), standard deviation (SD)
