# Supplementary material for: Community-level epidemiology of soil-transmitted helminths in the context of school-based deworming: Baseline results of a cluster randomised trial on the coast of Kenya
Source: PLoS Negl Trop Dis. 2019 Aug 9;13(8):e0007427. doi: 10.1371/journal.pntd.0007427 (PMC6719894; doi:10.1371/journal.pntd.0007427)
Supplement: S3 Table — (PDF) [file pntd.0007427.s006.pdf]

S3 Table. Individual- household- and environmental factors for individuals with matched parasitological data vs. those individuals without matched parasitological data across 120 clusters on the south coast of Kenya, 2015.

	Number with outcome available* N (%)	Number missing outcome[x] N (%)
**INDIVIDUAL FACTORS**		
**Sex**		
Male	7871 (40.0)	1316 (42.4)
Female	11811 (60.0)	1788 (57.6)
**Age**		
<5 years	1569 (8.0)	281 (9.1)
5-14 years	6066 (30.8)	830 (26.7)
≥15 years	12047 (61.2)	1993 (64.2)
**Attend school**		
No	12142 (61.7)	2027 (65.3)
Yes	7540 (38.3)	1077 (34.7)
**Received ALB (last 12months)**		
No	14566 (74.7)	2296 (75.1)
Yes	4921 (25.3)	763 (24.9)
**Observed shoe type**		
No Shoes	10824 (55.0)	1612 (52.1)
Shoes	8842 (45.0)	1480 (47.9)
**Open defecation§**		
Yes	10586 (53.9)	1604 (52.0)
No	9053 (46.1)	1481 (48.0)
**HOUSEHOLD FACTORS**		
**Household SES**		
1 (Poorest)	5997 (30.5)	940 (30.3)
2 (Middle)	9888 (50.2)	1521 (49.0)
3 (Least poor)	3797 (19.3)	643 (20.7)
**Household flooring**		
Earth/sand	15518 (78.9)	2333 (75.2)
Covered	4160 (21.1)	770 (24.8)
**Reported toilet facility access**		
None	9369 (47.6)	1401 (45.2)
Shared access	4613 (23.5)	783 (25.3)
Private access	5691 (28.9)	915 (29.5)
**Water Source**		
Non-Improved	9219 (47.0)	1287 (41.8)
Improved	10406 (53.0)	1795 (58.2)
**≤ 30 mins to water source**		
No	3812 (19.5)	550 (17.8)
Yes	15769 (80.5)	2533 (82.2)
**ENVIRONMENT FACTORS**		
**Urban/ rural**		
Rural	14682 (74.6)	2184 (70.4)
Periurban	3817 (19.4)	644 (20.7)
Urban	1183 (6.0)	276 (8.9)
**Aridity**		
Semi-arid	2355 (12.0)	331 (10.6)
Dry sub-humid	6064 (30.8)	772 (24.9)
Humid	11263 (57.2)	2001 (64.5)
**Altitude (metres)**		
Low (<59)	6510 (33.1)	1210 (39.0)
Medium (59-170)	6674 (33.9)	1003 (32.3)
High (>170)	6498 (33.0)	891 (28.7)
**EVI**		
Low (<0.3)	6481 (32.9)	930 (30.0)
Medium (0.3-0.4)	6552 (33.3)	1035 (33.3)
High (>0.4)	6649 (33.8)	1614 (36.7)
**Sand content of soil (%)**		
Low (<59)	4865 (24.7)	813 (26.2)
Medium (59-61)	8020 (40.7)	1208 (38.9)
High (>61)	6797 (34.5)	1083 (34.9)

**pH (KCl)**		
Low (<51)	4774 (24.3)	711 (22.9)
Medium (51-52)	8210 (41.7)	1414 (45.6)
High (>52)	6698 (34.0)	979 (31.5)

* 16,682 individuals included with STH data. The two outliers (one with hookworm intensity of 137,460epg and one with *T. trichiura* intensity of 99,804epg are excluded).

[x] 3104 individuals without STH outcome data

Acronyms: albendazole (ALB), confidence interval (CI), environmental vulnerability index (EVI), incidence rate ratio (IRR), odds ratio (OR), potassium chloride (KCL), socioeconomic status (SES), standard deviation (SD)
